# Testing Effects of Seed Treatments against Clubroot Disease in Various Oilseed Rape Hybrids

**DOI:** 10.3390/pathogens12111339

**Published:** 2023-11-10

**Authors:** A. Michael Klueken, Yamen Mahfoud, Sabine Rößler, Jutta Ludwig-Müller

**Affiliations:** 1 Bayer AG, Crop Science Division, Disease Control Biology, 40789 Monheim am Rhein, Germany; michael.klueken@bayer.com; 2 Faculty of Biology, Technische Universität Dresden, 01217 Dresden, Germany; yamen.mahfoud@tu-dresden.de (Y.M.); sabine.roessler@tu-dresden.de (S.R.)

**Keywords:** clubroot, isotianil, *Bacillus amyloquefaciens* QST713 HiCFU, oilseed rape, *Plasmodiophora brassicae*

## Abstract

Clubroot disease, caused by the protist pathogen *Plasmodiophora brassicae*, is an emerging threat to cruciferous crops, including oilseed rape (*Brassica napus* L.). Most of the current commercial cultivars are highly susceptible, and efficient management tools are lacking practical implementation. Over three years and three experimental periods, we studied the effects of isotianil in comparison with *Bacillus amyloliquefaciens* QST713-HiCFU against clubroot disease under greenhouse experiments. Our results show control effects, which were strongly dependent on seasons, host plant genotype, and clubroot isolates: isotianil and *B. amyloliquefaciens* QST713-HiCFU reduced disease severity consistently at variable, but field-relevant spore concentrations of clubroot isolates; with seed treatments showing superior effects compared to drench applications. The co-application of isotianil with *B. amyloliquefaciens* QST713-HiCFU could, in some cases, increase the efficacy. Interestingly, all studied hybrids reacted to treatments, albeit to a somewhat different extent. When tested against a field isolate, the results obtained with the single spore isolate were partially confirmed but with greater variability. Overall, the generally positive effects of isotianil and *B. amyloliquefaciens* QST713-HiCFU on the reduction of clubroot were repeatedly observed. The inoculation of clubroot disease with different spore counts indicates a dose–response effect for tested products. This study highlights the importance of performing experiments holistically over multiple, consecutive seasons, with various isolates, application types, and different genetic resources of host plants.

## 1. Introduction

Clubroot disease is caused by the obligate biotrophic protist *Plasmodiophora brassicae*. The disease is spread throughout the world and affects commercially important *Brassica* crops, including oilseed rape. The eukaryotic pathogen resides in soils and can infect suitable hosts from there. The life cycle is divided into two phases, and both could be targets for control mechanisms [1,2]. Clubroot disease can build up rapidly if susceptible hosts are present under conducive environmental conditions. Otherwise, the disease can also persist in soils as resting spores for up to or even more than 20 years when a suitable host is absent [3].

Despite resistant cultivars, effective practical control options are currently lacking. Clubroot disease is an emerging threat to cruciferous plants, including agriculturally important crops like oilseed rape (*Brassica napus* L.). Not only are most of the current commercial rapeseed cultivars highly susceptible, but resistant cultivars can be overcome by new virulent isolates of *P. brassicae* [4,5,6]. Such populations of new virulent isolates can spread rapidly, and effective strategies for long-term control are currently missing. The mechanism by which the pathogen breaks down resistance in plant cultivars is under discussion. It was found that many pathogens, such as *P. brassicae*, can deliver effectors that interact and suppress plant defenses; among them are enzymes such as methyl transferase, which derives the plant hormone salicylic acid [7]. Other effectors of *P. brassicae* possibly target earlier events, such as recognition by plant receptors [8]. The plant can recognize the effectors and mount a defense response, including various strategies depending on the respective pathogen [9]. To help the plant even in the presence of effectors, chemical or biocontrol control agents can induce such defense responses. In high-risk areas for *P. brassicae*, the current disease control strategies rely predominantly on a mix of tools with varying potentials to combat the disease (lime application of calcium ions, raising soil pH, irrigation in susceptible plant growth stages, etc.). Finally, effective biological or chemical plant protection products are lacking.

In their evolution, higher plants have developed various defense reactions to prevent pathogenic infections, e.g., through the formation of physical barriers on plants’ surfaces as well as through the development of various plant specific immune responses [10,11,12]. The activation of barriers and of plants’ specific elicitors has been demonstrated to be an effective way to protect plants from diseases. Researchers have more recently focused on both elements because they are regarded as a new and sustainable type of control strategy in regenerative agriculture. This is of particular interest for difficult-to-control pathogens, such as clubroot disease. Nowadays, there are many different plant protection products on the market that contain bacterial, fungal, and chemical ingredients. The fungicide resistance action committee (FRAC; [13]) has grouped various active ingredients according to their known modes of action. The group P “host plant defense induction” and the group BM “BM: Biologicals with multiple modes of action: Microbial” list several examples, including isotianil and *Bacillus amyloliquefaciens* strain QST713-HiCFU, respectively [13].

Plant-induced resistance can be classified into systemic acquired resistance (SAR) and induced systemic resistance (ISR), based on the molecular mechanisms of induction [12,14]. The gene expression pathways are studied, understood, and confirmed in some plants of agricultural importance [15,16]. For example, SAR is mediated by salicylic acid as the signaling phytohormone, while ISR utilizes jasmonic acid and ethylene [14].

Isotianil was discovered by Bayer AG in 1997 and later marketed for disease management in different countries around the world [17]. However, its particular mode of action was better understood only during the last decade. Bektas and Eulgem [18] as well as Portz et al. [19] showed that isotianil can act like an elicitor that mimics salicylic acid with significant activity against blast diseases in rice and wheat but without having a direct effect against these diseases. Similarly, isotianil can help to reduce difficult-to-control diseases like *Fusarium* wilt of banana caused by the fungal agent *Fusarium oxysporum f. sp. cubense*, including tropical race 4 [20]. The molecule belongs to the mode of action group “P03”, i.e., salicylate-related host plant defense induction [13].

The microbial inoculant *Bacillus amyloliquefaciens* strain QST713-HiCFU [21] is considered safe to humans [22] and is marketed by Bayer AG for soil application under various trade names, e.g., ‘Minuet’ in the USA. This formulation for seed treatment and soil use has been optimized for this specific purpose, containing a high count of particularly viable colony forming units (CFUs) for effective root colonization. The *Bacillus* strain QST713 has a strong ability to produce lipopeptides, such as surfactins, which are reportedly essential for biofilm and pellicle formation as well as the secondary root colonization process [23,24]. Moreover, important interactions with root exudates for host plant defense are considered to take place in the rhizosphere, where bacterial byproducts seem to be important [25,26,27]. Multiple studies have shown that these interactions can trigger SAR/ISR for various host pathogen interactions [28,29]. Subsequently, the FRAC committee reclassified QST713 from F6 (Code 44) in 2020 to “biologicals with multiple modes of action” (BM02).

The present study was conducted over a period of three years in a row and with three experimental periods in each year. To quantify the effect of isotianil in comparison with the *B. amyloliquefaciens* QST713-HiCFU formulation, disease severities and shoot performances were rated against several parameter combinations of the two control agents: three spore concentrations, two spore isolates, and six different *B. napus* cultivars/hybrids. Our results show control effects, which were strongly dependent on seasons, host plant genotype, and *P. brassicae* isolates.

## 2. Materials and Methods

### 2.1. Seed-Treated Hybrids/Plant Material

All winter oilseed rape (*Brassica napus*) cultivars or hybrids employed in this study are susceptible to *P. brassicae* and are described in Table 1; seed treatments are described in Table 2. The seeds were treated in the seed treatment facilities of Bayer AG in Monheim, Germany, using professional seed treatment equipment (batch treater type Niklas W.N. 5/01; Willy Niklas GmbH Apparatebau, Germany). The process of the seed treatment was carried out according to manufacturer information by mixing the products in water to apply a total slurry of 3 L 100 kg^−1^ to the seeds [30]. Twenty plants per treatment were used to improve reproducibility. A total of 3 independent replicates of 19 treatments were planned. The experiments were evaluated 7–8 weeks after inoculation by phenotyping (disease index and infection rate), taking pictures, and performing fresh weight determinations of the roots and shoots separately.

### 2.2. Pathogen Origin, Growth Media, Infection

Two different isolates of *Plasmodiophora brassicae* were used, one single spore isolate (SSI) e3 [31,32] and one field isolate characterized as 16/03/31 with the European Clubroot Differential [33]. The field isolate of *P. brassicae* was selected from a small collection at Technische Universität (TU) Dresden based on its performance on *Brassica rapa* ssp. *Pekinensis* (Chinese cabbage). The resting spores were isolated from mature Chinese cabbage galls, used for propagation. Three different spore concentrations of 10^6^, 10^7^, and 10^8^ *P. brassicae* spores mL^−1^ were used for inoculation. The field isolate came originally from agricultural land near the town of Leipzig, Germany, and was selected based on its high infectivity on Chinese cabbage; the exact isolates for the first experimental inoculation came from frozen (−20 °C) galls of Chinese cabbage, which had been stored at TU Dresden. The greenhouse experiments were carried out over a three-year period and with three separate replicates per year. For experiments 2 and 3 in the third year, the spores were taken directly from the galls of highly infected plants from the previous experiment.

Untreated seeds were pre-germinated on moist filter paper in glass Petri dishes under room conditions for 3 days (for the control and drench treatments). Emerging *Brassica* seedlings were planted in 13 × 13 × 13 cm pots (2 seedlings/pot) filled with soil (Einheitserde Classic Standard soil type P, pH 5.8, Hermina Samen, Germany), which was sieved coarsely and mixed with sand (Sahara Spielsand, Hornbach, Germany) with a soil/sand ratio of 4:1. Then they were hot-steam sterilized for 120 min after cooling down and adequately watered prior to sowing, and put onto a tray with water. Coated seeds were sown directly in the same soil mixture in the pots. Plants grew in the greenhouse with a temperature range from 16 to 29 °C and relative humidity between 19 and 75% (Figure S1). Plants were irrigated two times a week with tap water, but no fertilizer was used.

Rapeseed plants were infected five days after treatment with isotianil and *B. amyloliquefaciens* QST713-HiCFU (10 days after sowing) with 2 mL of inoculum per plant (Table 1 and Table 2). The *P. brassicae* spore solution was pipetted to each seedling (2 mL). In addition, a control treated with 2 mL phosphate buffer only (50 mM KH_2_PO_4_, pH 5.5) was performed. In each treatment, 30 plants were used at a density of two plants per pot. Across various experimental repetitions, three concentrations of *P. brassicae* were used (10^6^, 10^7^, 10^8^), in addition to a control treated with 2 mL phosphate buffer (50 mM KH_2_PO_4_, pH 5.5). Isotianil and *B. amyloliquefaciens* QST713-HiCFU were diluted for the drench treatments with dH_2_O. For drench treatments, the compounds were applied close to the base of the plant as a 2 mL solution. For drench treatments, the same concentrations of active ingredient (a.i.) as for the seed treatments were used (Table 2). All experiments were conducted under greenhouse conditions documented in Figure S1, and the disease was quantified using various parameters.

### 2.3. Disease Documentation

In all studies, the disease severity of the upper plant parts were visually assessed over time and photographed for documentation. The experiments were evaluated 7–8 weeks after inoculation by destructive sampling to loosen the soil, carefully pulling out the root system and shaking the plant gently until all excess soil was removed. Subsequent assessments included phenotyping (disease index and infection rate), pictures, and fresh weight determination of the roots and shoots separately.

For the evaluation of disease severity, three different factors were evaluated [34]: (1) the infection rate; (2) the disease index that indicates the severity of the disease (additionally, the numbers of plants in the individual disease classes are shown); (3) the shoot fresh weight is the value that shows how much better the upper plant parts are performing. The disease classes were as follows: 0 = no symptoms, 1 = very small swellings at the lateral roots, 2 = larger swellings at the lateral roots, 3 = larger galls found also at the main roots, but some parts of root system still present, 4 = transformation of the complete root into a gall. The disease index was calculated using the following equation:

DI = (1 × Cl1 + 2 × Cl2 + 3 × Cl3 + 4 × Cl4) + 100/4 × number infected plants

For the heat map, the efficacy of treatments for reducing clubroot symptoms was calculated using the following equation:

Efficacy = 100 − (T/C × 100),

where T is the treatment and C the control group with *P. brassicae* only.

The biomass indicators can provide evidence of reversing the general status of plants in the treatment, and they are positively related to the expected yield and maturity indices. The shoot index was calculated for all treatments according to the non-infected control, which is the weight of the infected/control shoots.

### 2.4. Statistical Analyses

The analysis of variance for the obtained data was performed with GenStat^®^ software (version 12), using the analysis of variance (ANOVA) test. The mean separation was analyzed using Duncan’s multiple range test (LSR) at 5% probability, because the number of treatments was more than five. The individual datasets are either given in the main manuscript or shown in the Supplementary Materials.

## 3. Results

### 3.1. Product Performances Following Different Application Techniques

The aim of this study was to investigate the effects of two agents that induce plant defenses, isotianil and *Bacillus amyloliquefaciens* strain QST713-HiCFU, in relation to clubroot disease (Table 1). Based on previous experiences, we chose a long-term study design in which the experiments were performed over a period of three years using six different *B. napus* hybrids and two *P. brassicae* isolates. In each experiment, clubroot disease was successfully established, progressing over time with the development of typical symptoms.

**Figure 1:**
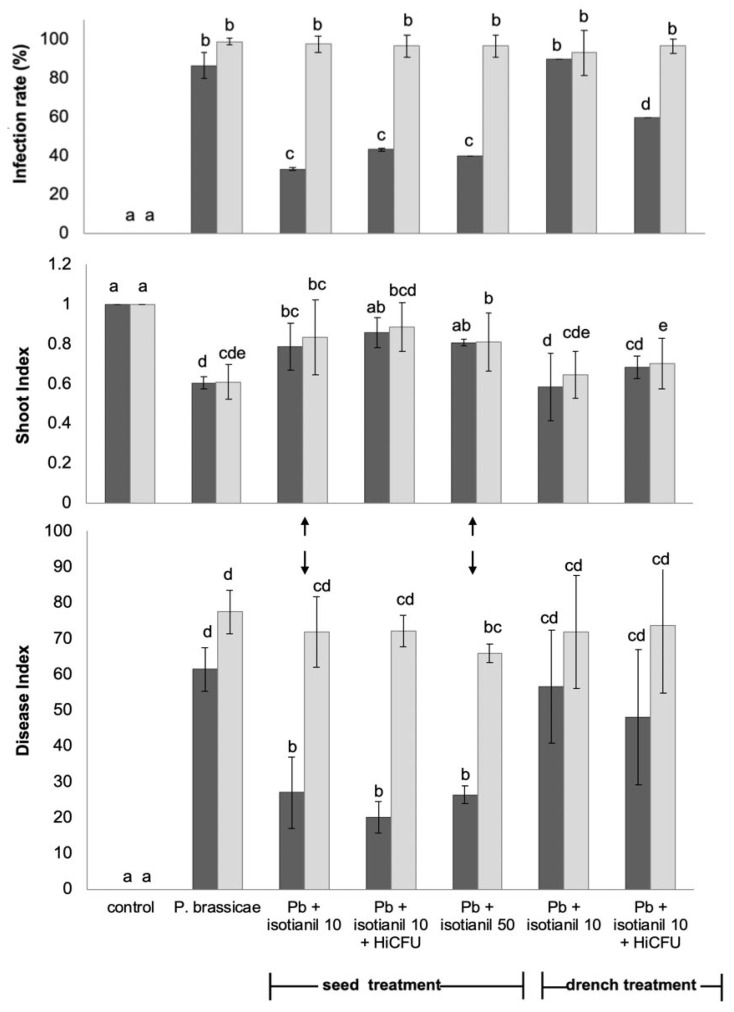
Comparison of different drench and seed treatments with the cv. ‘Jenifer’. Control = no treatments; Pb = *P. brassicae* = inoculation with 10^6^ or 10^7^ spores mL^−1^ (dark grey and light grey, respectively); isotianil applied at 2 concentrations (indicated by the 2 arrows) and with/without HiCFU = *Bacillus amyloliquefaciens* QST713-HiCFU. The concentrations and methods for seed coating are given in the methods. Mean values for 3 individual experiments, including 20 plants per treatment, are shown. The statistically significant differences for *p* ≤ 0.5 are indicated by different letters per inoculation density.

**Figure 2:**
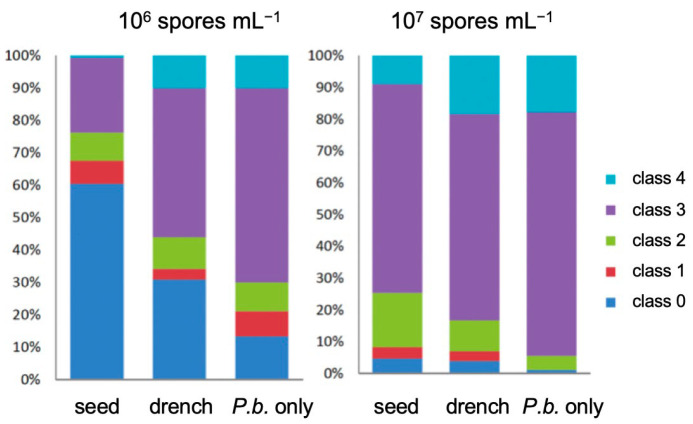
Mean percentage of plants in individual disease classes at *P. brassicae* (*P.b.*) 10^6^ and 10^7^ spores mL^−1^ for all drench vs. seed treatments using cv. ‘Jenifer’. Examples of root pictures in the different disease classes are shown in Figure S2.

Two different forms of application, drench vs. seed treatments, together with two different isotianil concentrations, were tested in the first experiment with hybrid cv. ‘Jenifer’ and the single-spore isolate e3 (Figure 1 and Figure 2). The majority of treatments resulted in a reduction in disease severity, as determined by the infection rate, the disease index (DI), and the shoot index (Figure 1). The latter shows the growth performance of the plant, with a high shoot index, indicating that plants are overall healthier than those with a low shoot index. A high DI indicates highly susceptible plants, whereas a low DI stands for tolerant plants. Specifically, class 1 has very small swellings at the lateral roots, class 2 has larger swellings at the lateral roots, class 3 shows already larger galls at the main and lateral roots, and class 4 means that the complete root was transformed into a large gall and necrotic tissue occurred, too. Class 0 indicates for non-infected roots. Some examples of the classes and what galls look like in different hybrids are shown in Figure S2.

With the respective spore concentrations of *P. brassicae*, we could achieve, in hybrid ‘Jenifer’, an infection rate of more than 90%, which was well suited to the experimental needs. Seeds treated with isotianil had a DI of 26 compared to the controls, which were between DI 70 and DI 80 (Figure 1). Generally, the seed treatments performed better than the drench treatments, but higher isotianil concentrations did not result in lower disease severity (Figure 1, arrow). Drench treatments had a lower DI than the infected control, but the differences between their roots were not statistically significant. In contrast, roots growing from treated seeds compared to those from untreated seeds revealed statistically significant differences in the DI; within these comparisons, the difference between the two isotianil concentrations was not significant. The lowest DIs were initially obtained when seeds were treated with both isotianil and *B. amyloliquefaciens* QST713-HiCFU. However, this combination was not repeated in the subsequent experiments. In addition, the influence of the degree of infection on the performance of test products was observed; at low spore concentrations, the infection rate was clearly lower for all isotianil treatments, but at high spore concentrations, all products were significantly less effective or even ineffective. The exception to this general tendency was the shoot index data, which continued to show positive plant health gains from the use of isotianil as a seed treatment, even under high disease pressure. Moreover, in those controlled environment trials, where pathogen pressure was moderate, all compounds reduced clubroot severity, particularly when applied as seed treatments, but they were not as effective when pathogen pressure was higher.

To calculate the severity of the infection, the percentage of each category in the infection scale is displayed (Figure 2). While the disease index provides an estimate of the total severity based on all classes, the percentage data can directly indicate how many plants are in one class, allowing for better assessment of disease severity. More plants in classes 0 (→ no infection; blue) and 1–2 (→ mild infection; red and green) indicates more tolerance, while higher numbers in classes 3 and 4 (violet and cyan) denotes susceptibility. The high numbers in classes 3 and 4 indicates a strong infection, as displayed for treatments with *P. brassicae* only, which can be found in the first column of each panel. The proportion of plants in lower disease classes was higher in the different seed treatments. Likewise, these data indicate a better performance of the seed treatments of *B. napus* compared to the drench treatment at both *P. brassicae* spore concentrations. Therefore, the seed treatments at low concentrations were selected to further investigate the effect on other commercial hybrids of *B. napus*.

### 3.2. The Effect of Isotianil Is Primarily Dependent on Hybrid, P. brassicae Isolate, Spore Concentration, and the Season

**Figure 3:**
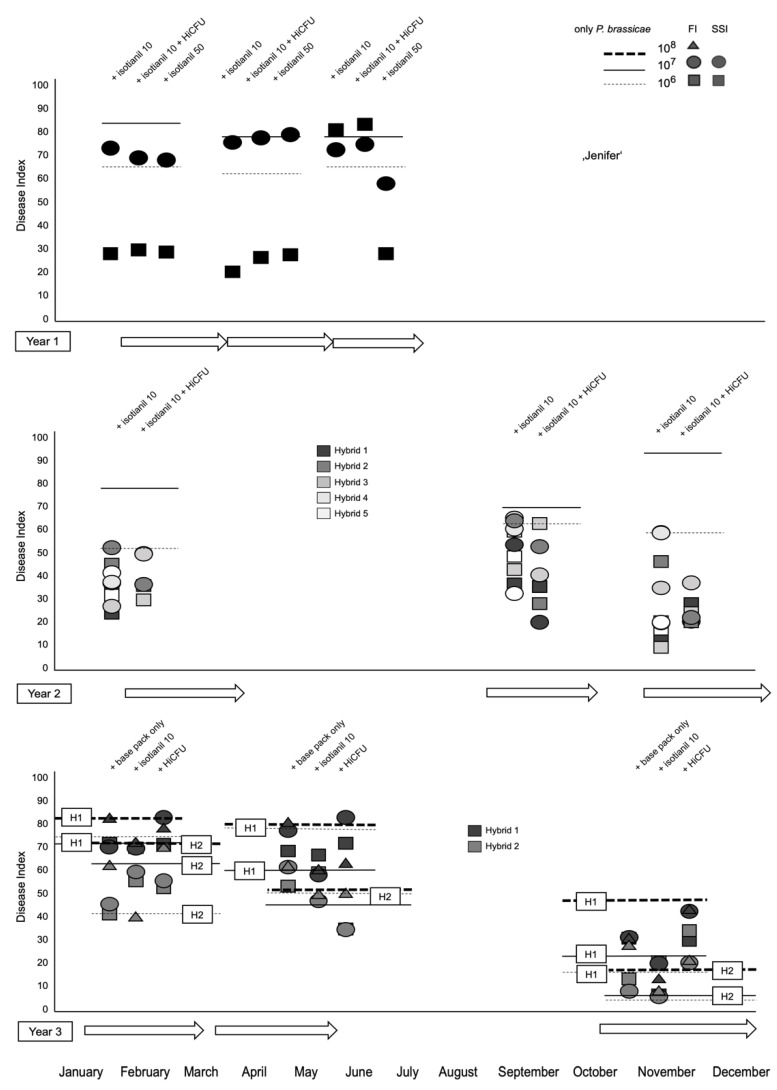
Disease indices are shown for the experimental years 1, 2, and 3 (top-down) as well as for 3 experimental units per year (related to the seasons, left–right) and descriptions (oblique) per year for treatments above the data points. The treatments with *P. brassicae* are only depicted as (dashed) lines for all spore concentrations (description top right in Figure), whereas the round, square, and triangle shapes indicate these concentrations for the hybrids (hybrid ‘Jenifer’ in year 1, hybrids 1 to 5 in year 2, and hybrids 1 and 2 in year 3), and the origin of *P. brassicae* isolates (FI = field isolate; SSI = single spore isolate). In years 1 and 2, the SSI, and in year 3, the FI were used for inoculation. The individual datasets for the other parameters are shown in the Supplementary Materials (Figure S3), and the mean values for the second and third years are shown in Figure 4. For the details of the experimental periods, see also Table 2. Base pack/coat = coating solution containing fungicide base package as seed treatment only.

We noted a strong influence of season on the experiments conducted over the three-year period (Figure 3). Therefore, the chosen study design proved to be effective. Even though the variation between experiments (based on untreated but inoculated plants) was greater than the observed treatment differences within experiments, it was still demonstrated overall that isotianil treatments reduced disease severity. This effect was increased in some hybrids by adding *B. amyloliquefaciens* QST713-HiCFU. No treatment resulted in severe growth inhibition of the shoot parts (Figure S5). The mean values indicate better performances for almost all hybrids tested as already seen for the susceptible variety, ‘Jenifer’. Although all experiments were carried out in the greenhouse under semi-controlled conditions (Figure S1), additional factors during the different periods may have affected the performance. A summary of all growth conditions can be found in Table 1.

**Figure 4:**
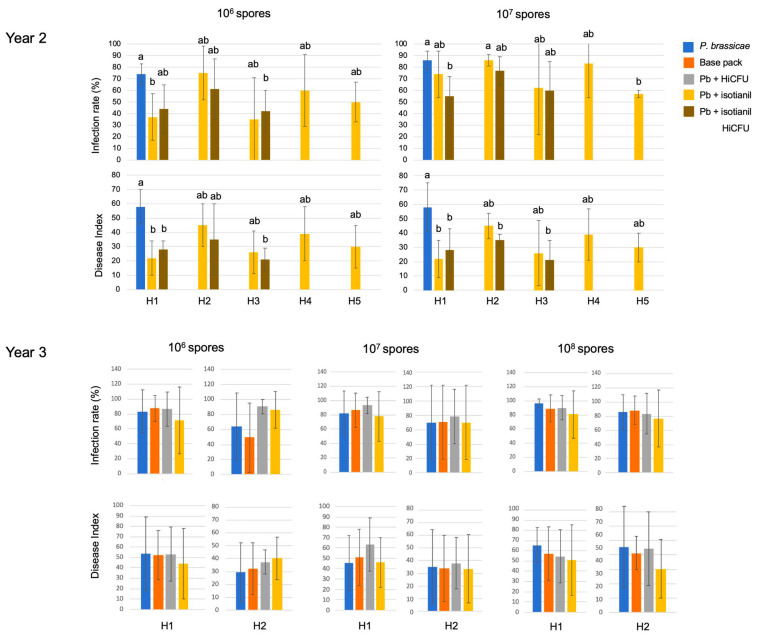
Root disease parameters for year 2 with single spore isolate e3 (upper panels) and year 3 with the field isolate (lower panels). In year 2, *P. brassicae* infection, concomitant treatment with isotianil and isotianil together with HiCFU were compared. In year 3, additionally, the coating solution alone was used, and instead of the combination treatment of the two compounds, only HiCFU was investigated. The information on hybrids can be seen in Table 1. The mean values for 3 individual experiments including 20 plants per treatment are shown. The statistically significant differences for *p* ≤ 0.5 are indicated by different letters per inoculation density. The color key can be found in the top right of the figure. HiCFU = *Bacillus amyloliquefaciens* QST713-HiCFU; base pack/coat = coating solution containing fungicide base package as seed treatment only.

At first glance, the conditions in the greenhouse during the three-year experimental period do not appear to be that variable, nevertheless, there seems to be an effect on growth and performance from the different testing periods (Figure S1). The humidity varied across different experimental time points but was similar for all three experiments. The temperature variations were largest in experiment 3 and lowest in experiment 1. Some other parameters varied among the experimental years, too. We, therefore, decided to present the data for all hybrids over the season but separated according to the experimental years (Figure 3). This representation indicates that most treatments had an effect on reducing disease severity, although the more consistent effects were obtained for the lower spore concentrations in the first, but for higher spore concentrations in the second year. All data summaries can be found in the Supplementary Materials when they are not presented in the main manuscript (Figures S3 and S4). The infection rates at lower spore concentrations (presumably occurring under natural conditions) were not substantially different (in observed intensity) from those produced at higher spore concentrations (>10^7^ spores mL^−1^) in the greenhouse tests. However, there could be an underlying influence on efficacy, too. Therefore, a higher concentration of 10^8^ spores mL^−1^ was also tested in one experimental period. Based on these data, it seems that the combined treatment of isotianil and of *B. amyloliquefaciens* QST713-HiCFU might have resulted in a better outcome for some hybrids (Figure 3). Compared to the results obtained in the first year, the reduction using isotianil persisted at lower spore concentrations, although not as effectively as originally observed (Figure 3 and Figure 4).

The aim of the third experimental set was to test higher spore concentrations and to compare a field isolate to the SSI. All spore concentrations used are believed to occur under natural conditions. The field isolate was selected based on its performance in recent experiments on Chinese cabbage. In general, the galls had a more furrowed appearance compared to the ’rounder’ shape caused by the SSI e3 (Figure S2). The field isolate used in the first experiment was from a frozen stock at the TU Dresden laboratory. The next two experiments were carried out with fresh spores that were generated from the previous experiments on oilseed rape plants. This could have led to the observed differences in disease expression, i.e., the latter two fresh isolates were possibly more aggressive, but using the same stock solution for all experiments was not possible. In all treatments of experiment 3, the infection rate was 100% and only for the lowest spore concentration of 10^6^ was it below 100% in one replicate. However, relatively large differences were observed for the hybrids, treatments, and spore concentrations (Figure S3). For this experiment, the infection rate for the isotianil treatments of hybrid 1 was always lower, and for hybrid 2, even at the high spore concentration of 10^8^. The *B. amyloliquefaciens* QST713-HiCFU treatments alone did not result in a reduction of clubroot infection. The isotianil treatment of hybrid 1 led to a significant reduction in the DI for low and high spore concentrations, although this was not consistently found over all concentrations, since at 10^7^ spores per ml, it did not show a difference (Figure 3). For hybrid 2, isotianil only reduced disease severity at the highest spore concentration in the first experiment. In the second experiment, a reduction using isotianil was found only for hybrid 2 at the highest spore concentration, and in experiment 3, there were no large differences found in general. In this representation, the effects of the individual seasons per experimental year become visible.

Again, we calculated for these two experimental periods the percentage of plants in the individual disease classes (Figures S3 and S4). In the second experimental year, the percentages of plants in individual disease classes showed more variation, but at least in two experimental replications at low spore concentrations, the isotianil treatment reduced the number of highly diseased plants for some hybrids (Figure S3C). For the third experimental year, the results for the individual disease classes were not so clear (Figure S4C). The fungicide-based seed treatment alone was tested only in one year. This resulted in some changes compared to the untreated controls, but this effect was also not consistent across hybrids and clubroot spore concentrations. When administered alone, the *B. amyloliquefaciens* QST713-HiCFU treatments were not more effective compared to their combination with isotianil (Figure 3 and Figure 4). The calculation of the mean values (Figure 4) showed that the effect was valid across all experiments; however, individual variations are reflected in high error bars, and the differences were not statistically significant in all cases. Based on these overall data, it seems that hybrids 1 and 3, and possibly partially hybrid 5, performed better than hybrids 2 and 4, when inoculated with the SSI (Figure 4 and Figure S3). However, for the field isolate, for which only two hybrids were tested, a positive effect of isotianil was only found under variable conditions for one of the two hybrids, and mainly in experiment 1. However, treatments with *B. amyloliquefaciens* QST713-HiCFU did not show any reduction at all (Figure S4A,B).

### 3.3. Shoot Growth Is Not Consistently Affected

Shoot growth was obviously not affected by treatments, and neither growth inhibition nor growth promotion could be detected. All plants were still green at the end of all experiments in all three testing periods. Regarding the influence on upper plant parts, the shoot weights of the control and infected plants were recorded per disease class. Subsequently, the ratio of “infected vs. controls” was calculated (shoot index). A higher shoot index means that the plants performed better even though they were infected. Alternatively, the shoot weight can also be used as trait.

**Figure 5:**
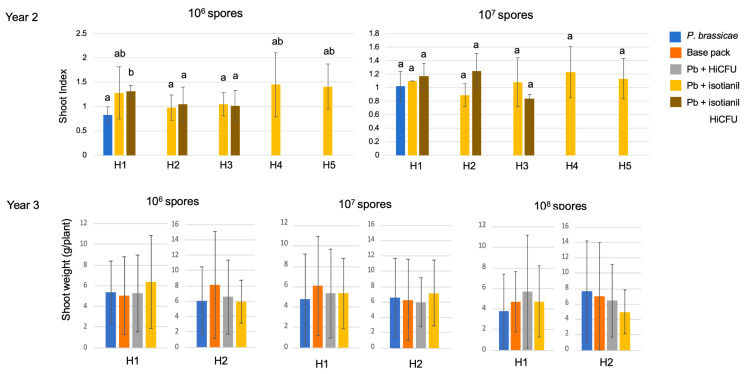
Shoot phenotype is given as the mean shoot index for the experiments for year 2 with single spore isolate e3 (upper panel) and mean biomass (g/plant) for year 3 with the field isolate (lower panels). In year 2, *P. brassicae* infection, concomitant treatment with isotianil, and isotianil together with HiCFU were compared. In year 3, additionally, the coating solution alone was used, and instead of the combination treatment of the two compounds, only HiCFU was investigated. The information on hybrids can be seen in Table 1. The mean values for 3 individual experiments including 20 plants per treatment are shown. The statistically significant differences for *p* ≤ 0.5 are indicated by different letters per inoculation density. The color key can be found on the top right of the figure. HiCFU = *Bacillus amyloliquefaciens* QST713-HiCFU; coat/base pack = coating solution containing fungicide base package as seed treatment only. Photos for leaf phenotypes for year 3 with all treatments can be found in the Supplementary Materials (Figure S4).

While in the first experiment, a slight increase in the shoot index was found for the isotianil seed treatments (Figure 1), this could not be verified in the later experiments (Figure 5). The biomass, either determined as the fresh weight or shoot index (the latter by the ratio of fresh weight infected to control shoots), was not significantly altered within the second and third experimental periods (Figure 5). Pictures of the upper plant parts for various experiments do not show differences between the control and isotianil treatments (as an example, the complete set of the experimental year 3, with all treatments and organism combinations, is shown in Figure S5). For the other hybrids and inoculation with the SSI, no visible differences were found. It can be noted that the lower spore concentration of 10^6^ spores mL^−1^ had a less dramatic effect on the leaf phenotypes of both hybrids, but also in all treatments. The differences among the experiments are also visible. In experiments 2 and 3, more yellow to brownish leaves were found (Figure S5). The shoot weight (Figure 5) was expected to be higher for treated plants if they were performing better, but this was not consistently found in all experiments. The shoot weight per plant varied among the three experiments (Figures S3 and S4), but there was no discernible trend within a single experiment for a specific treatment. However, it would be worth taking a look at crop yields, as they could still make a significant difference following an application.

## 4. Discussion

### 4.1. Infection-Related Changes in Response to Host Plants

**Figure 6:**
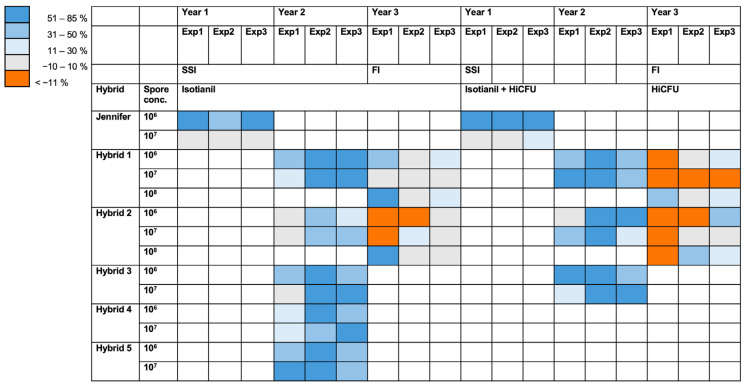
Heat map for disease indices of all experiments. To obtain the control effects for the different treatments, the efficacy was calculated as described in the Materials and Methods section. This was translated into a scale, with three blue shades for positive control effects and one for negative effects. Efficacies below 10 were not considered and are depicted in grey. Only seed treatments are shown for isotianil, *Bacillus amyloquefaciens* QST713-HiCFU in comparison to control treatments. No color = hybrid/cultivar not tested; grey = no change; shades of blue = positive (control) effect of treatment compared to *P. brassicae* only; orange = (much) higher in treatment than control; SSI = single spore isolate; FI = field isolate.

Clubroot disease is still difficult to control with measures that would be environmentally and/or economically acceptable, and therefore, the majority of cultural measures often involve the growth of resistant cultivars, although the resistance is often overcome by new and more aggressive *P. brassicae* isolates [4,5,6]. In the present study, alternative treatments were tested in greenhouse trials. The infection parameters were reduced after treatments with the plant defense-inducing agents isotianil and *B. amyloliquefaciens* QST713-HiCFU. This effect was dependent on the mode of application (where seed treatments performed superiorly to drench application), the type of *B. napus* hybrid, the *P. brassicae* spore concentration, and also the pathogen isolate. In addition, a strong seasonal effect was noted. For better comparisons, a heat map representing all disease indices is shown in Figure 6. To obtain a better visualization of the achieved effects, the control effectiveness was calculated based on the disease indices. A trend can be observed in the isotianil treatments, particularly for experimental year 2. Auer and Ludwig-Müller [35] reviewed the control efficacies of various biocontrol agents against clubroot and found that the majority of studies reported a reduction in clubroot symptoms following treatments with biological control agents (BCA). Interestingly, some studies reported efficacies with more severe symptoms observed after BCA treatment. This is similar to what we found when using the field isolate in the third experimental year after treatments with isotianil or with *B. amyloliquefaciens* QST713-HiCFU.

In the context of the overall experimental set-up, a number of different treatment variables were investigated in the first tests; here, one hybrid and two different spore concentrations of the SSI e3 were tested. It was found that the seed treatments performed better than the drench treatments for isotianil and for *B. amyloliquefaciens* QST713-HiCFU (Figure 1). Drench treatments had a lower DI than the infected control, but the differences were not significant. However, they were found to be significantly different when comparing the roots from treated seeds with those from untreated seeds. Contrary to this, Peng et al. [36] reported that for a commercial *Gliocladium* biocontrol formulation, the drench treatment performed better than a seed treatment. Therefore, the most efficient way to use compounds or biocontrol agents to combat a particular disease must always be verified in specific experiments.

The biocontrol agent, *B. amyloliquefaciens* QST713, has been developed on various crops for foliar applications mainly, including oilseed rape, and is already globally marketed as Serenade [37,38]. A new formulation of this agent has been optimized for performance in the soil environment, when used as seed treatment or under drench application, and the effects against clubroot disease are clearly visible, as demonstrated in the current study. So, it can be assumed that the mode of action has been improved but not changed. In this sense, Lahlali et al. [39,40] found that mechanistically, the determination of the expression of selected defense genes pointed to the activation of JA- and ET-related defense pathways in *B. napus*. A similar mechanism to induce defense is associated with the compound isotianil [17,18,19]. Therefore, we could show that both control agents are potential new tools to reduce clubroot disease in oilseed rape.

Surely, after infection with clubroot pathogens on roots, the whole plant is affected. This also includes effects on upper plant parts, which are often the root cause of negative yields [37,38]. The experiments were not suitable for determining the effects of isotianil and *B. amyloliquefaciens* QST713-HiCFU on yield parameters. Thus, studies in this direction could provide valuable insights to complete the overall picture of both management tools.

### 4.2. The Environment Can Influence Disease Severity

Environmental conditions could also be involved in the outcome of an infection with *P. brassicae*. It is known that temperature has a strong effect on the infection success of *P. brassicae* when invading different host plants, including *B. napus* [36,41,42]. An optimum temperature is required for maximum infection; below or above this limit, infection rates decline [36]. Therefore, variations in temperature that exceed the optimum may result in reduced efficiency in colonizing host roots. Also, the relative humidity in the air can have an effect on infection, but it is less pronounced. The relative humidity is usually controlled in growth chambers and greenhouse conditions between 60 to 80% [6,43,44], but a possible effect of this variation has not been researched yet. Moreover, the soil moisture plays a larger role per se [45,46,47] since the pathogenic zoospores are motile and need to find host roots over some range in the soil [48]. As a consequence, the agriculture production of oilseed rape could become more difficult, and it could become harder to achieve consistent yield levels over many years. So, farmers may like to use products like isotianil or *B. amyloquefaciens* QST713-HiCFU in combination with tolerant or resistant hybrids to successfully keep diverse management tools alive to successfully reduce clubroot disease.

## 5. Conclusions

Our study demonstrates that (1) isotianil treatment reduced clubroot severity at two different spore concentrations, a severe level and a moderate level, both of which can be found under field conditions; (2) co-application with *Bacillus amyloquefaciens* QST713-HiCFU could, in some cases, increase the benefit; (3) a strong seasonal effect was observed; and (4) all hybrids used in this study reacted to the treatments, although to a somewhat different extent. Therefore, it would also be of interest to add more clubroot resistant cultivars to such a setup. The data obtained with the field isolate only partially confirm the data previously obtained with a single spore isolate because they are more variable. The season in which each experiment was carried out influenced the outcome of the experimental data to a certain degree. However, the generally underlying positive effects on the reduction of clubroot were independent of the year of testing. The inoculation with different spore counts indicates that the efficacy of the test product was related to the inoculum load; it is important to note that very high concentrations of inoculum do not obviously lead to more infection in untreated controls; this has been observed at least in our greenhouse experiments. Similarly, very low inoculum concentrations do not lead to high infection rates anyway; therefore, further reduction may be indistinguishable from the treatments. All treatments, including those with *P. brassicae* only, did not result in direct changes to upper plant parts.

However, the yield could also be affected, which was not tested in our study setup. Understanding yield impacts related to pathogen infection, seed treatments, and host plant hybrids would require experiments either in the field or closer to field conditions, where plants are grown for a longer time. Experiments to simulate more natural field conditions are, therefore, needed in the future. Since a single spore isolate of *P. brassicae* was used, it is recommended that future studies include a more representative mix, containing both laboratory and field isolates. Moreover, growth conditions need to be more adapted to field conditions: first, soil from more natural conditions can be introduced, and second, the experimental conditions should switch from pots with two plants each to large containers. This would allow for a more natural simulation, if *P. brassicae* resting spores were mixed into soils rather than infesting individual plants. It would also be of interest to determine whether the treatments combined with *P. brassicae* have an impact on the yield of oilseed rape if the plants could be grown over a longer period of time.

## Figures and Tables

**Table 1:** Overview of study timings and *Brassica napus* resources used.

Experimental Year	Experiment	Time Frame and Duration	No. of Plants/Treatment	Hybrid Name	Seed Source and Harvest Year	Seed Type	TKW (g) *	Germination
1	1a	6.3.2019–24.4.2019	30	Jenifer (4407)	Bayer AG, Oilseed Rape Centre, Monheim, 2018	Conventional Hybrid ^1^	4.92	98
	1b	27.3.2019–21.5.2019						
	1c	3.4.2019–4.6.2019						
2	2a	10.9.2020–30.10.2020	20	Hybrid 1 Hybrid 2 Hybrid 3 Hybrid 4 Hybrid 5	Bayer SAS Boissay France, 2020	Conventional Hybrid Conventional Hybrid Conventional Hybrid HOLL Hybrid ^2^ Dwarf Hybrid ^3^	4.91 4.51 5.90 6.68 5.43	98 100 92 91 96
	2b	26.11.2020–19.1.2021						
	2c	10.2.2021–8.4.2021						
3	3a	28.10.2021–6.1.2022	20	Hybrid 1 Hybrid 2	Bayer SAS Boissay France, 2020	Conventional Hybrid Conventional Hybrid	4.91 4.51	98 100
	3b	14.1.2022–17.3.2022						
	3c	25.3.2022–17.5.2022						

* Thousand kernel weight. ^1^ Standard hybrid with no special oil profile or any other special characteristics, achieved by conventional breeding methods; ^2^ HOLL stands for hybrid with high oleic, low linolenic fatty acid oil profile. This profile typically involves a few mutation events in the genome to alter the oil/acid profiles, and these mutation events are achieved by conventional breeding methods. ^3^ Dwarf or Semi-dwarf hybrids have shorter plant height than conventional hybrids, but otherwise no marked difference in metabolite profile.

**Table 2:** Treatment list per study time and experiment. The *P. brassicae* isolates were e3 (single spore = SSI) and a field isolate (FI) with ECD code 16/03/31. HiCFU = *Bacillus amyloliquefaciens* QST713-HiCFU; ST = seed treatment; drench = drench application at planting.

Study Period	Treatments	Formulation	Dose Rates	Application	*P. brassicae* Inoculum
Year 1	Untreated non-inoculated	-	-	-	-
	2. Untreated	-	-	-	SSI
	3. Isotianil	* FS200	0.1 g/kg	ST	SSI
	4. Isotianil and HiCFU	FS200 and FS150	0.1 g/kg and 6.6 mL/kg	ST	SSI
	5. Isotianil	FS200	0.5 g/kg	ST	SSI
	6. Isotianil	FS200	0.5 g/ha	drench	SSI
	7. HiCFU	FS150	5 L/ha	drench	SSI
Year 2	Untreated non-inoculated	-	-	-	-
	2. Untreated	-	-	-	SSI
	3. Isotianil	FS200	0.1 g/kg	ST	SSI
	4. Isotianil and HiCFU	FS200 and FS150	0.1 g/kg and 6.6 mL/kg	ST	SSI
Year 3	Untreated non-inoculated	-	-	-	-
	2. Untreated	-	-	-	FI
	3. ** Coat	-	-	ST	FI
	4. Coat and Isotianil	and FS200	and 0.1 g/kg	ST	FI
	5. Coat and HiCFU	and FS150	and 6.6 mL/kg	ST	FI

* Formulation strength: Products were applied as suspension concentrates for seed treatment (FS): with, e.g., FS200 containing 20% and FS150 15% of active material, formulated as flowable concentrate for seed treatments, respectively. ** Coat/base pack = coating solution containing fungicide base package as seed treatment only.

## Data Availability

The datasets supporting the conclusions of this article are either presented in the main text of the manuscript or available in the Supplementary Materials as individual panels.

## Supplementary Materials

The following supporting information can be downloaded at: https://www.mdpi.com/article/10.3390/pathogens12111339/s1, Figure S1: Greenhouse parameters over the three experimental periods.; Figure S2: Examples for the different disease classes for all hybrids with SSI e3; Figure S3: Individual data for experiments of year 2; Figure S4: Individual data for experiments of year 3; Figure S5: Shoot phenotypes of with *P. brassicae* inoculated seeds from control and isotianil treatments from the third-year experiment.
