# Supplementary material for: Testing Effects of Seed Treatments against Clubroot Disease in Various Oilseed Rape Hybrids
Source: Pathogens. 2023 Nov 10;12(11):1339. doi: 10.3390/pathogens12111339 (PMC10675021; doi:10.3390/pathogens12111339)
Supplement: Supplementary file 1 [file pathogens-12-01339-s001.zip › pathogens-2663549-supplementary.pdf]

Supplement to
"Testing effects of seed treatments against clubroot disease in various oilseed rape hybrids"

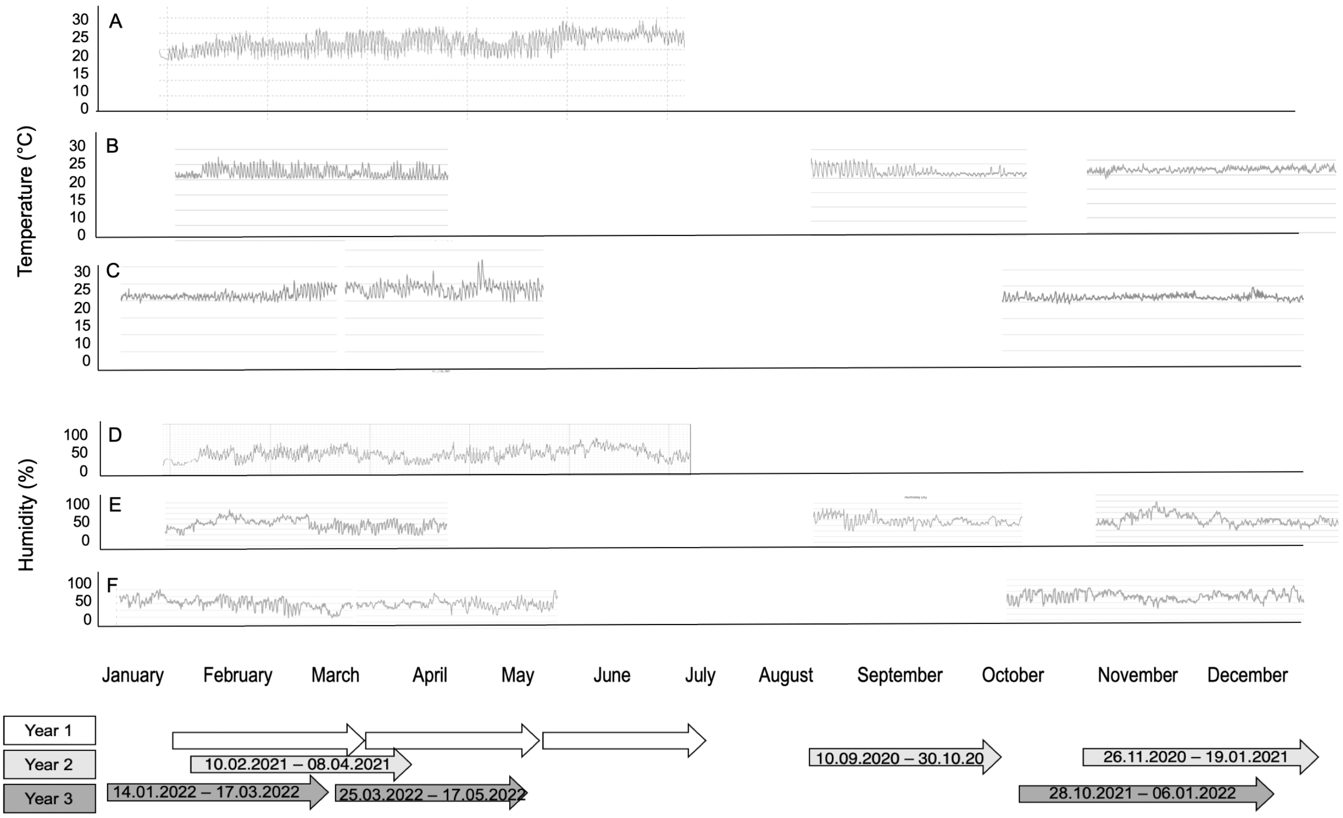

Figure S1. Greenhouse parameters over the three experimental periods. A, D: Year 1; B, E: year 2; C, F: year 3.

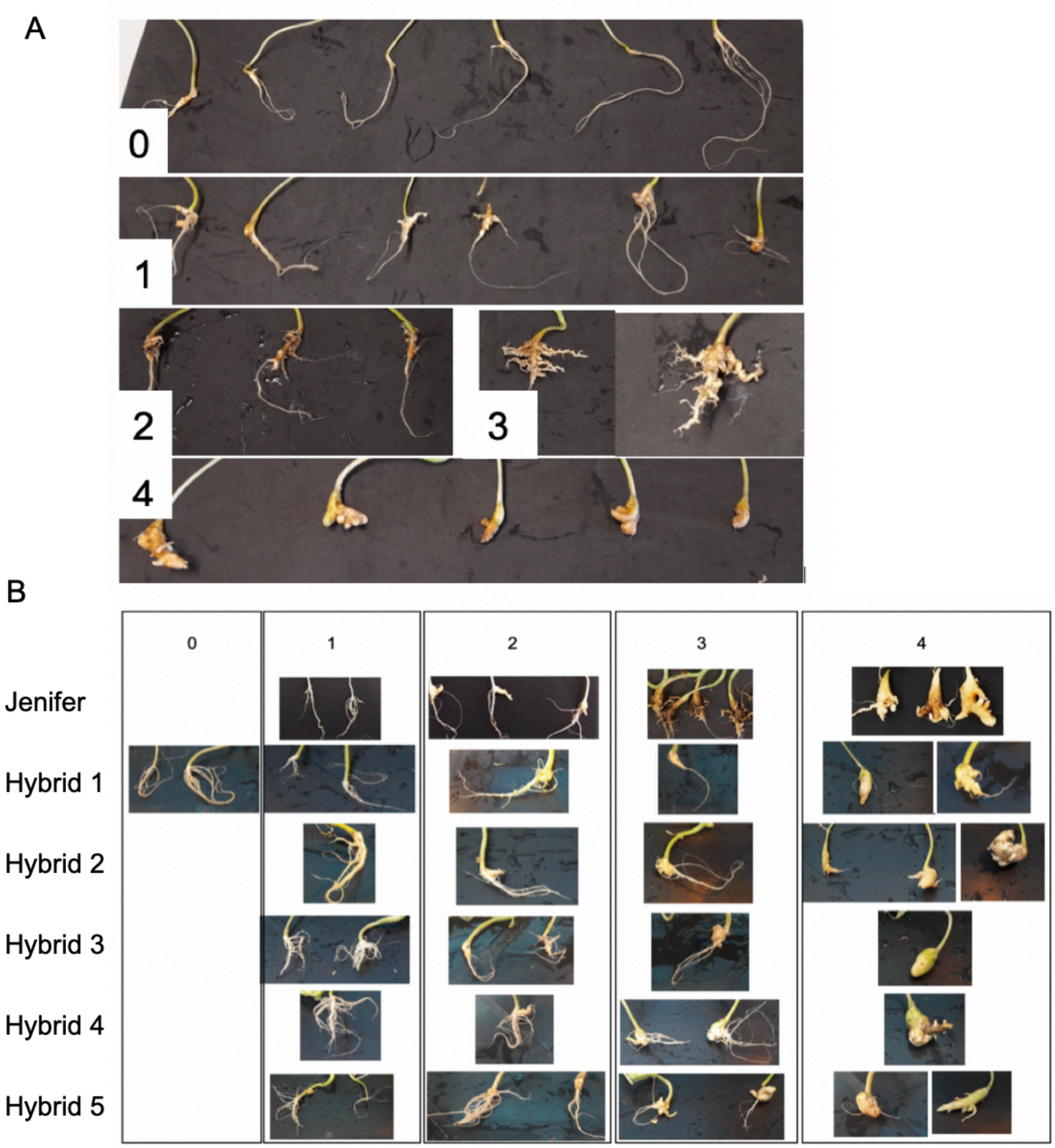

Figure S2. A. Examples for the different disease classes indicated in the photos by 0 - 4 with the field isolate at a spore concentration of 10^6 mL^{-1} on hybrid 1; B. Examples of different classes with SSI e3 and all hybrids used in this study at a spore concentration of 10^6 mL^{-1}. Clubs of different hybrids with e3 SSI do not show different phenotypes.

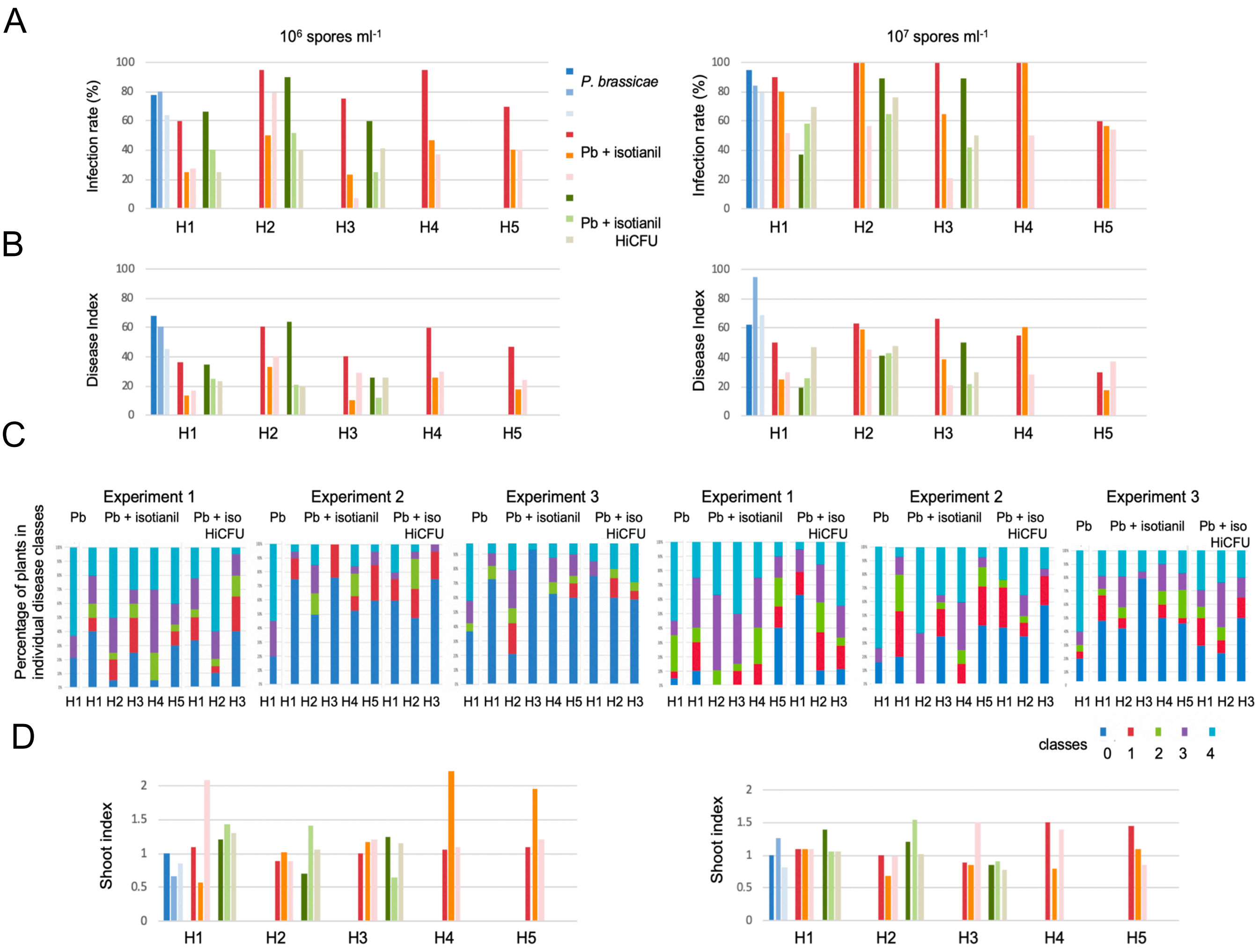

Figure S3. Data from year 2. *P. brassicae* (Pb) isolate e3 10^6 spores/ml and 10^7 spores/ml as well as different hybrids (H1 – H5) and treatments (I = Isotianil; *Bacillus* = *Bacillus amyloquefaciens* QST713 HiCFU); Different shades represent the individual experiments in one year. A. Infection rate; B. Disease Index; C: Percentage of plants in individual disease classes and the scale goes from zero to 100 %; D. Shoot index. The color key can be found between and below the panels.

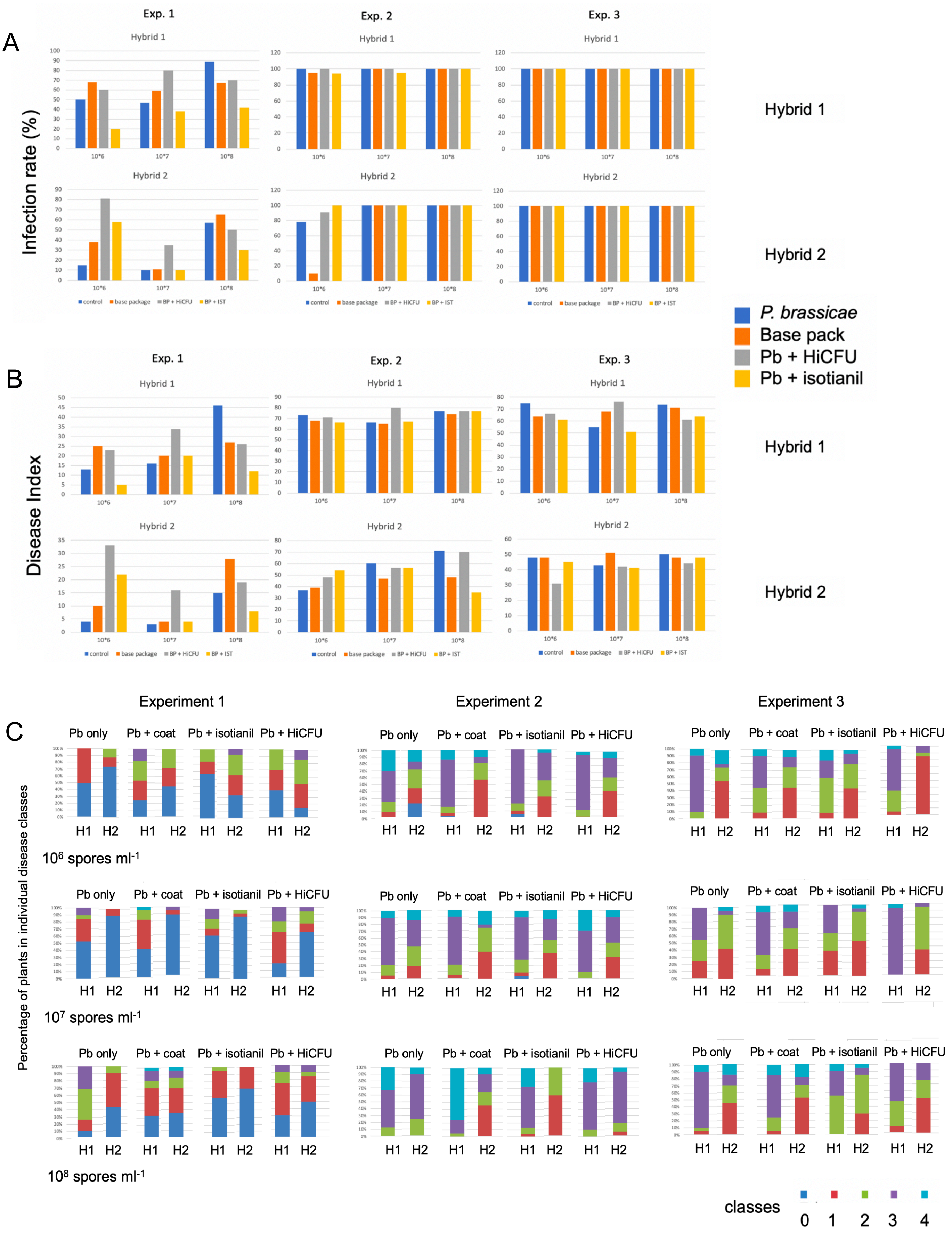
A
B
C
Exp. 1
Exp. 2
Exp. 3
Hybrid 1
Hybrid 2
Infection rate (%)
Disease Index
control
base package
BP + HiCFU
BP + IST
10^6
10^7
10^8
P. brassicae
Base pack
Pb + HiCFU
Pb + isotianil
Experiment 1
Experiment 2
Experiment 3
Pb only
Pb + coat
Pb + isotianil
Pb + HiCFU
H1
H2
Percentage of plants in individual disease classes
10⁶ spores ml⁻¹
10⁷ spores ml⁻¹
10⁸ spores ml⁻¹
classes
0
1
2
3
4

D

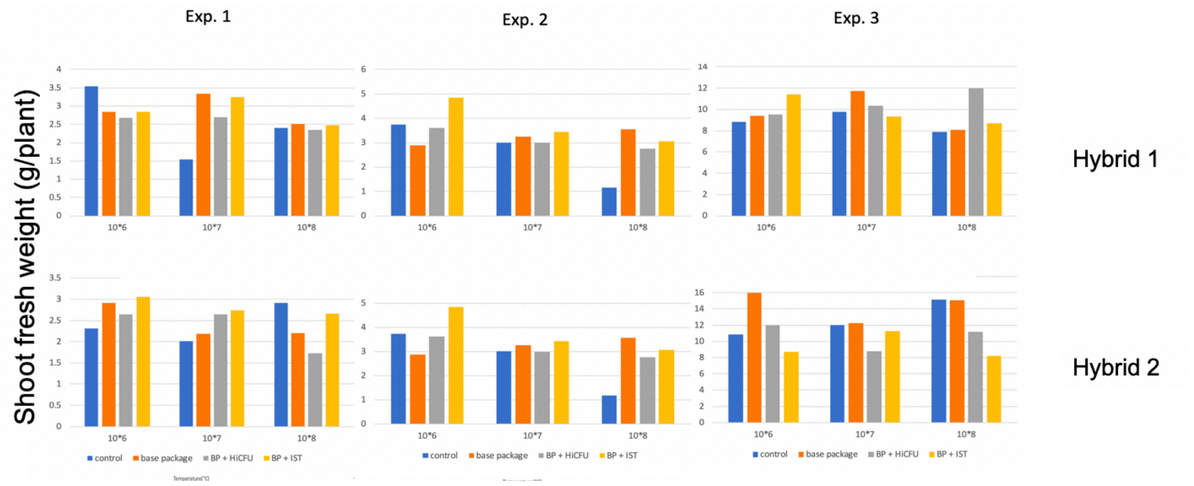

Figure S4. Data from year 3: *P. brassicae* (Pb) field isolate 10^6 spores/ml, 10^7 spores/ml and 10^8 spores/ml as well as different hybrids (H1, H2) and treatments (I = Isotianil; *Bacillus* = *Bacillus amyloquefaciens* QST713 HiCFU). The three different experiments (exp) are shown separately, for mean values see Figure 4. A. Infection rate; B. Disease Index; C: Percentage of plants in individual disease classes; D. Shoot weight/plant. The color key can be found between and below the panels.

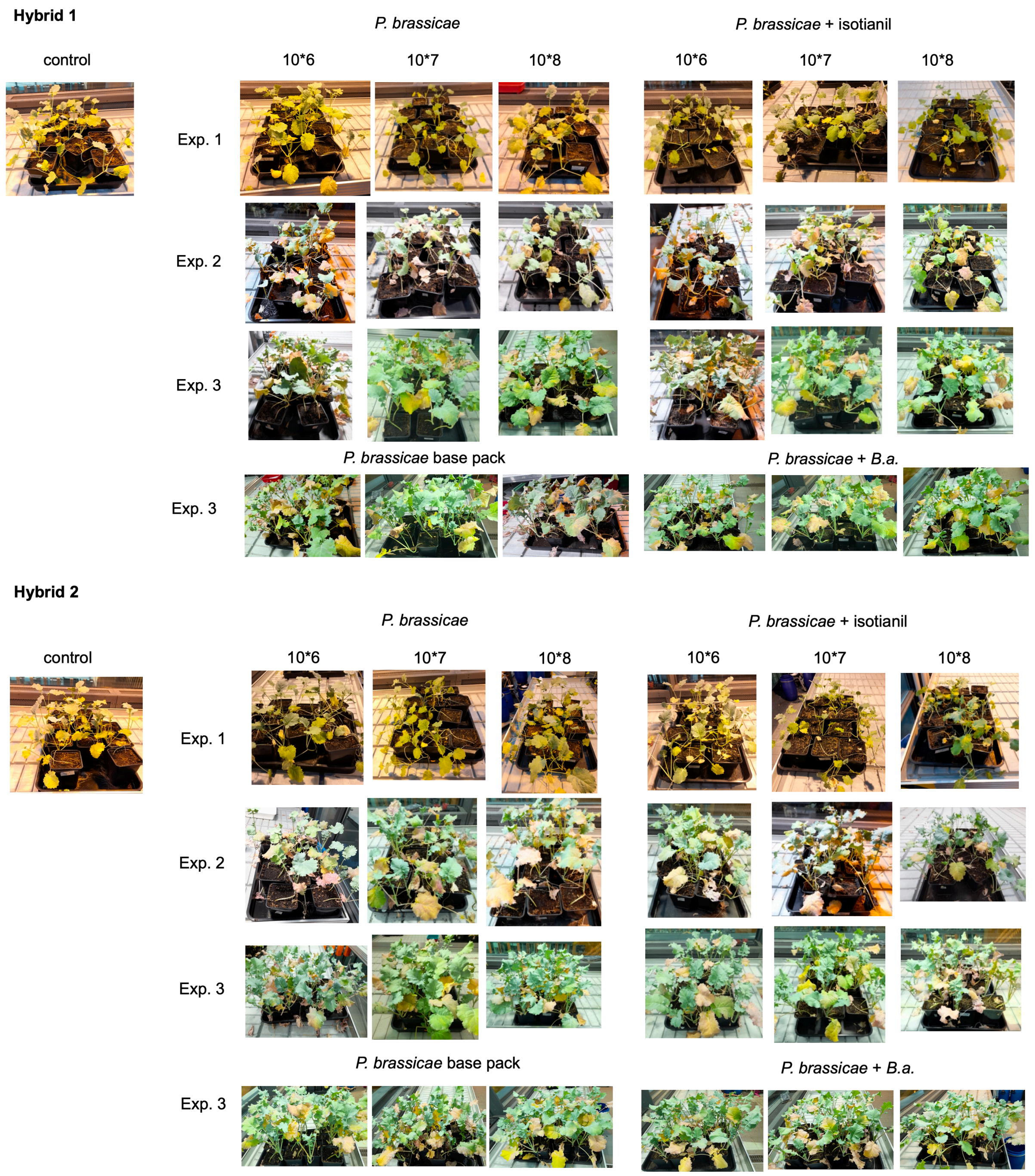

Figure S5. Shoot phenotypes of with *P. brassicae* inoculated seeds from control and isotianil treatments from the third-year experiment. Upper panel shows the pictures for hybrid 1, the lower panel for hybrid 2, both inoculated with the field isolate. All three experiments indicate no difference between leaf phenotypes. For one third-year experiment (Exp. 3) also the leaves for Bacillus treated plants (B.a.) only and base pack (coating solution only) are shown.
